# Advances in intravenous thrombolysis with tenecteplase in the ultra-time window

**DOI:** 10.3389/fneur.2026.1724311

**Published:** 2026-02-18

**Authors:** Zhen Wang, Hong-jian Guan, Lin-zhuo Qu, Qi Han, Yong Wang, Chun-hua Quan

**Affiliations:** 1 Department of Neurology, the Affiliated Hospital of Yanbian University (Yanbian Hospital), Yanji, China; 2 Yanbian University School of Medicine, Yanji, China; 3 Central Laboratory, the Affiliated Hospital of Yanbian University (Yanbian Hospital), Yanji, China

**Keywords:** acute ischemic stroke, intravenous thrombolysis, ischemic penumbra, tenecteplase, time window

## Abstract

Worldwide, stroke has become a significant public health concern, with ischemic strokes accounting for over 70% among all stroke types. Intravenous thrombolysis (IVT), as a traditional treatment method, is limited by a narrow 4.5-h time window, which restricts its application. The conception of a “tissue window” for imaging evaluation has prompted a change in treatment strategy in recent years, and the time window has been gradually expanded to 24 h. Tenecteplase (TNK), a third-generation thrombolytic medication with a long half-life, good fibrin specificity, and minimal risk of bleeding, has garnered significant research interest in ultra-time window thrombolysis. TNK is noninferior to alteplase (rt-PA) at 4.5 h and has a superior reperfusion rate in patients with large vessel occlusion in the anterior circulation, according to several phase III randomized controlled studies (e.g., AcT, TRACE-2, ORIGINAL). Ultra-time window studies (e.g., TRACE-3 and TIMELESS) have demonstrated that TNK has a positive safety profile and substantially enhances functional outcomes in patients who possess an ischemic penumbra detected by imaging screening for 4.5–24 h. Nevertheless, certain research (such as the CHABLIS-T II study) demonstrated that the application of ultra-time window intravenous thrombolysis improves reperfusion but doesn't improve the prognosis. Therefore, multicenter trials, coupled with neuroprotective medications or anti-inflammatory therapy, and dynamic imaging stratification are required to optimize intravenous thrombolysis strategies. This article serves as a reference for clinical practice and research direction by conducting a systematic review of the recent findings on ultra-time window thrombolysis in TNK.

## 1 Introduction

As one of the major causes of death and disability in adults, stroke has become a major public health problem worldwide, resulting in a heavy disease burden (1). Currently, China has the highest rate of stroke in the world (2), with ischemic stroke accounting for over 70% (3–5). It is crucial to open the occluded blood vessels as soon as possible to reperfuse the ischemic area. A primary therapeutic approach for treating acute ischemic stroke (AIS) is intravenous thrombolysis; however, due to the conventional 4.5-h window, just a handful of individuals are admitted to the hospital in time for thrombolysis (2). The investigation of thrombolysis extended time window based on imaging assessment (e.g., perfusion-core mismatch, DWI-FLAIR mismatch) has advanced recently, and the time window for intravenous thrombolysis has been expanded to 24 h. TNK, a genetically modified variant of rt-PA, emerges as a promising candidate for thrombolysis in the extended time window. Its favorable pharmacological profile—including a longer half-life, higher fibrin specificity, reduced systemic fibrinogenolysis, and bolus administration—addresses several limitations of alteplase. In this article, we provide a comprehensive overview of the most recent TNK research progress in patients with AIS in the ultra-time window and look forward to future therapeutic strategies.

## 2 Dynamic evolution and imaging assessment of the ischemic penumbra

### 2.1 Dynamic evolution of the ischemic penumbra

In 1981, ASTRUP et al. first proposed the concept of ischemic penumbra (6). This region of brain tissue, characterized by cerebral blood flow between 10 and 20 mL/(100 g·min), is dynamic and progressively contracts with prolonged ischemia, potentially evolving into the irreversibly damaged infarct core (7). By restoring perfusion to the ischemic penumbra and restoring it promptly, further aggravation of brain damage can be prevented. Therefore, restoring blood supply to the ischemic penumbra as soon as possible is crucial to the treatment of AIS patients (8). From a pathophysiological perspective, the ischemic penumbra vanishes, and the majority of the damage is irreversible when the brain tissue experiences ischemia and hypoxia for longer than 4.5 h. The conventional time window for IVT has been defined at 4.5 h following the onset of the illness in accordance with this pathophysiological process. Although the ischemia time and ischemia velocity influence the size of the ischemic penumbra, the traditional time window only focuses on the ischemic time and does not take into account the individual variability in infarct progression (9, 10).

### 2.2 Imaging-guided clinical decisions

Many factors influence the dynamic evolution of the ischemic penumbra (11, 12). The compensatory capacity of collateral circulation is a crucial factor that determines the ischemic area and the spatial distribution size of ischemic penumbra (13). Additionally, in reperfusion therapy, the compensatory ability of collateral circulation serves as an assessment metric for the rates of revascularization and hemorrhage conversion. In addition to increasing the efficacy of therapy, the DEFUSE-3 and DAWN studies showed that endovascular therapy guided by imaging technologies extended the length of time between 16 and 24 h (14, 15). Subsequently, the time span was extended from 9 h to 24 h by the TRACE-III research and the EXTEND study (16, 17). Consequently, the tissue window has replaced the traditional time window as the study direction. With advancements in imaging technology, the ischemic penumbra can now be clinically examined by examining collateral circulation status, clinical symptom-imaging mismatch, and imaging mismatch. For assessing whether AIS patients who have passed the temporal window can benefit from reperfusion therapy, this has emerged as a critical criterion (9–11). Research shows that substituting a tissue window for the conventional time window as the criterion for intravenous thrombolysis eligibility can increase thrombolysis eligibility to about 30% of patients with post-awakening stroke (18), which will make it easier to choose reperfusion therapy for AIS patients who awaken after onset or whose onset time is unknown.

## 3 Pharmacologic benefits and clinical evidence for tenecteplase

### 3.1 Pharmacological properties

As a genetically modified version of rt-PA, TNK is a third-generation intravenous thrombolytic medication that optimizes fibrin specificity, increases plasma half-life, and enhances resistance to Plasminogen Activator Inhibitor-1 (PAI-1) by substituting the amino acids at sites 296–299 by alanine, asparagine by glutamine at site 117, and threonine by asparagine at site 103. Due to the benefits of exclusively working on fibrin at the site of thrombosis, the ease of administration, and the potent PAI-1 resistance, the danger of systemic hyperfibrinolysis and bleeding can be reduced (19). When it comes to pharmacokinetics, TNK is mainly metabolized by the liver and interacts with more blood clots since it has a longer half-life and lower plasma clearance compared with rt-PA (20). Based on these characteristics, it is a suitable medicine for ultra-time window thrombolysis.

### 3.2 Non-inferiority and dose optimization

The NOR-TEST trial is the first phase III effectiveness trial to examine TNK, demonstrating that following treatment with 0.40 mg/kg TNK and 0.9 mg/kg rt-PA, respectively, for IVT-eligible AIS patients, the modified Rankin Scale (mRS) score did not differ significantly in the proportion of <2 points or the incidence of adverse events within 24–48 h. Both trials also had similar safety outcomes, indicating that TNK was not superior to rt-PA (21). Because the NOR-TEST trial included several patients with mild strokes, the natural prognosis was more favorable, which concealed the superiority of TNK.The NOR-TEST 2, part A study (22) indicated that 0.40 mg/kg of TNK had a higher risk of bleeding than 0.9 mg/kg of rt-PA (21% vs. 7%, *P* = 0.006), as well as a tendency toward a higher risk of symptomatic intracranial hemorrhage (sICH) and a poor prognosis, which was stopped early. As a result, the TNK dosage was changed to 0.25 mg/kg for the NOR-TEST 2, Part B research that followed. In a meta-analysis of four randomized controlled trials, Katsanos et al. (23) demonstrated that TNK was superior to rt-PA for AIS patients with concurrent large vessel occlusion during the thrombolytic time window in terms of the percentage of patients who achieved an mRS score of 0–2 at 3 months, the rate of successful vascular recanalization, and the probability of functional improvement.

In recent years, a total of five phase III RCTs collectively established that TNK (0.25 mg/kg, max 25 mg) is non-inferior to rt-PA (0.9 mg/kg, max 90 mg) for AIS within 4.5 h, with comparable safety profiles (24–28) (Table 1). With an increasing number of large RCTs, a meta-analysis that included three studies—NOR-TEST, AcT, and TRACE-2—reaffirmed that 0.25 mg/kg TNK is more effective than 0.9 mg/kg alteplase in treating AIS (29). In addition, two studies, including one in a Chinese population, reported lower rates of symptomatic bleeding with TNK compared to rt-PA (30, 31).

**Table 1:** Comparison of phase III RCTs of TNK vs. rt-PA for acute ischemic stroke.

**Study (Year)**	**Sample size (intervention/ control)**	**Intervention/ control (mg/kg)**	**mRS score 0–1 at 90 days (%)**	Safety outcome	
				**Incidence of sICH (%)**	**mortality rate (%)**
AcT (24) (2022)	1577 (806/771)	TNK: 0.25 vs. rt-PA: 0.9	36.9 vs. 34.8	3.4 vs. 3.2	15.3 vs. 15.4
TRACE-2 (25) (2023)	1407 (705/696)	TNK: 0.25 vs. rt-PA: 0.9	62 vs. 58	2 vs. 2	7 vs. 5
ORIGINAL (26) (2024)	1465 (732/733)	TNK: 0.25 vs. rt-PA: 0.9	72.7 vs.70.3	1.2 vs. 1.2	4.6 vs. 5.8
ATTEST- II (27) (2024)	1777 (885/892)	TNK: 0.25 vs. rt-PA: 0.9	44 vs. 42	2 vs. 2	8 vs. 8
TASTE (28) (2024)	680 (339/341)	TNK: 0.25 vs. rt-PA: 0.9	57 vs. 55	3 vs. 2	7 vs. 4

AcT, Comparison of intravenous thrombolysis with TNK vs. rt-PA in patients with acute ischemic stroke; TRACE-2, Comparison of TNK vs. rt-PA for acute ischemic cerebrovascular events; ORIGINAL, Comparison of the effectiveness of TNK vs. rt-PA for acute ischemic stroke; ATTEST-II, TNK vs. rt-PA in AIS patients within 4.5 h of onset; TASTE, TNK vs. rt-PA in AIS patients chosen based on perfusion imaging.

The updated Guidelines for the Early Management of Acute Ischemic Stroke by the American Heart Association and the Stroke Association in 2019 (32), the Guidelines for Intravenous Thrombolysis for Acute Ischemic Stroke by the European Stroke Organization (ESO) in 2021 (33), the Chinese Expert Consensus for the Treatment of Acute Ischemic Stroke with Tenecteplase published in China in 2022 (34), and the revised National Stroke Clinical Guidelines of the United Kingdom and Ireland by the National Institute for Health and Clinical Excellence in 2023 (35) all demonstrate how the recommendations for TNK in pertinent clinical guidelines are continuously updated and optimized in light of the results of multiple RCTs. In 2024, the Chinese Stroke Association issued a new guideline that recommended intravenous thrombolysis with TNK or rt-PA should be administered to AIS patients with a National Institutes of Health Stroke Scale (NIHSS) score of ≥4 if the disease onset occurs within 4.5 h (Class I recommendation, Level A evidence) (36). Given the research background mentioned above, thrombolytic therapy within an ultra-time window has become a more important consideration in clinical decision-making for AIS.

## 4 Research and breakthrough in ultra-time window intravenous thrombolysis

### 4.1 Ultra-Time window IVT for AIS with unknown onset time or wake-up stroke

According to national recommendations, the first-line treatment choice for AIS patients who are eligible for thrombolysis and arrive at the hospital within the traditional 4.5-h window is thrombolysis with TNK. Nonetheless, patients with an undetermined onset time (onset after awakening or unclear onset time) and ultra-time window frequently miss the opportunity for intravenous thrombolysis owing to the thrombolysis time window limitation. In the field of reperfusion, increasing the number of patients who can benefit from intravenous thrombolysis is therefore a hot topic at the moment.

By extending the IVT time window to 9 h under multimodal imaging screening, the EXTEND study (16), published in 2019, increased the number of IVT beneficiaries and laid the foundation for future research on ultra-window therapy for TNK. The WAKE-UP study (37) was chosen for AIS patients with waking stroke or AIS with unknown onset time and no mechanical thrombolysis (>4.5 h from the last known well (LKW) time but within 4.5 h from the time of symptom discovery), in which the head MRI plain scanning showed mismatches between diffusion-weighted imaging and the fluid-attenuated inversion recovery sequences. This study differed from the EXTEND study in that it focused on patients with unknown onset time and confirmed that thrombolysis could be beneficial for this patient group. Subsequent post hoc analysis of this experiment revealed that individuals with high FLAIR-rSI in the DWI lesion but no significant FLAIR high signal had less benefit from IV thrombolysis (38). Based on a multimodal image-guided ultra-time window RCT trial, four studies—WAKE UP, EXTEND, THAWS, and ECASS-IV—were compiled in a high-quality meta-analysis in 2020. In randomized controlled trials (RCTs), all included study participants were divided into two groups: one for intravenous thrombolysis and the other for a placebo control. Data analysis revealed that the 90-day good prognosis rate (mRS score 0–1) was higher in the intravenous thrombolysis group than in the placebo group (47% vs. 39%, *OR* = 1.49, 95% CI: 1.10–2.03, *P* = 0.011). Yet, the rate of bleeding adverse events was higher in the intravenous thrombolysis group than in the placebo group, but this did not impact the patients' benefit from the treatment (39). The TWIST study (40) aimed to investigate the efficacy of TNK treatment in patients with waking strokes (detected neurological deficits within 4.5 h) based on CT scanning screening. (1:1) to 0.25 mg/kg in the TNK-treated and placebo-controlled groups, and the results showed that the TNK-treated group did not demonstrate superior neurological outcomes (*aOR* = 1.18, 95% CI: 0.88–1.58, *P* = 0.27), but the difference in the 3-month adverse event rate between the two groups was not statistically significant. TNK intravenous thrombolysis is not advised for patients with waking stroke who were screened solely by cranial CT plain scanning, according to this study, which found that treatment did not improve the 90-day functional prognosis or lower the risk of death and intracranial hemorrhage. The TIMELESS (41) study enrolled 458 AIS patients with onset between 4.5 and 24 h. Concurrent CT and MRI perfusion images revealed the presence of intracranial anterior circulation macrovascular lesions and ischemic penumbra (infarct core volume <70 ml, mismatch ratio ≥1.8, mismatch volume ≥15 ml), and pre-treatment NIHSS scores >5 in adult AIS patients, of whom 77.3% underwent intravenous thrombolysis and bridging endovascular thrombolysis. The findings demonstrated that the TNK group did not outperform the placebo group in terms of improving the 90-day good functional outcome (mRS score 0–2) in patients with AIS. Additionally, there were no statistically significant differences between the two groups in terms of the incidence of sICH within 36 h or 90-day mortality (both *P* > 0.05). The independent thrombolytic effect may have been attenuated by a high fraction of bridging thrombolysis, which may be the reason why this trial did not support TNK's advantage. Still, it suggests its safety within the 4.5–24 h time window (42). The ROSE-TNK study (43) used the imaging criterion of DWI-FLAIR mismatch to evaluate AIS patients with onset of 4.5–24 h and concurrent blockage or severe stenosis of the relevant artery for intravenous thrombolysis. Patients were screened for intravenous thrombolysis using DWI-FLAIR-matched imaging criteria, which included simultaneous occlusion or severe stenosis of the accountable artery in AIS. The study also verified the safety of TNK thrombolysis within the 4.5–24 h time window.

The available research addresses the issue of heterogeneity in the effectiveness of ultra-temporal window IVT in AIS with unknown awakening or onset time. While multimodal imaging screens (e.g., EXTEND, WAKE-UP studies) confirm that thrombolysis within 4.5–9 h improves neurological prognosis in certain patients (e.g., mismatched volume attainment or DWI-FLAIR mismatch), other studies (e.g., TWIST, TIMELESS) have demonstrated that TNK thrombolysis did not significantly improve 90-day functional outcome in patients with a 4.5–24 h window who were screened solely by conventional CT or in combination with a high percentage of endovascular thrombolysis. This variation could result from a complex interplay between safety concerns (higher but statistically unstable variations in the incidence of sICH), combined therapeutic interferences (e.g., endovascular interventions attenuating the independent effect of thrombolysis), and patient screening criteria (type of imaging technique, differences in mismatch thresholds). To balance efficacy and safety and accomplish precision treatment, future research into the synergistic processes of independent thrombolysis and bridging therapy, as well as additional improvement of imaging stratification criteria, will be necessary.

### 4.2 Ultra-time window IVT for AIS with non-small vessel occlusion

Researchers have concentrated on using multicenter, large-sample clinical trials to assess TNK's effectiveness in promoting recanalization of occluded or severely stenotic vessels to confirm its capacity to achieve a higher recanalization rate and a higher rate of reperfusion for patients with non-small vessel occlusive AIS with onset time >4.5 h. Although several research findings have been published in recent years (Table 2), introducing fresh perspectives and difficulties to the field, the effectiveness remains debatable because of the variety of imaging screening techniques, the impact of varying administration timing, drug dosage, and patient type. The odd occurrence of reperfusion-prognosis disconnection stands out among them; it is thought to be connected to reperfusion injury or variations in collateral circulation, and its precise mechanism requires more investigation.

**Table 2:** Selected studies of ultra-time window IVT in AIS with non-small vessel occlusion.

**Study (year)**	**Sample size (TNK/control)**	**Time Window (*h*)**	**Patient type**	**Intervention/ control (mg/kg)**	**mRS score 0–1 at 90 days (%)**	Safety outcome	
						**Incidence of sICH (%)**	**mortality rate (%)**
CHABLIS-T (48) (2024)	86 (43/43)	4.5–24	LVO or near occlusion	TNK: 0.25 vs. TNK: 0.32	27.9 vs. 48.8	9.3 vs. 9.3	14.0 vs. 4.7
TEMPO 2 (49) (2024)	886 (432/454)	0–12	Mild stroke caused by LVO	TNK: 0.25 vs. SOC	69 vs. 71	2 vs. 0.5	5 vs. 1
CHABLIS-T II (50) (2025)	224 (111/113)	0–24	LVO, MVO	TNK: 0.25 vs. SOC	39.6 vs. 36.3	4.5 vs. 6.2	10.6 vs. 10.6

CHABLIS-T, Intravenous TNK thrombolysis in stroke patients selected by perfusion imaging within 24 h of onset; TEMPO 2, TNK vs. standard therapy for mild stroke with large vessel occlusion or near-occlusion; CHABLIS-T II, Intravenous TNK thrombolysis in stroke patients selected by perfusion imaging within 24 h of onset II; LVO, Large Vessel Occlusion; MVO, Medium Vessel Occlusion; SOC, Standard of Care (typically antiplatelet therapy).

As the first TNK trial to achieve positive results in the field of AIS treatment in the ultra-temporal window, the TRACE-3 study (17), a prospective, multicenter, randomized controlled, open-label, endpoint-blind evaluated phase III clinical study headed by Prof. Wang Yongjun's team at our university, offers crucial high-level evidence in this area. The following were the primary inclusion criteria: 1) onset of 4.5–24 h (including wake-up stroke and unwitnessed stroke); 2) occlusion of a large artery in the anterior circulation with an infarct core-low perfusion mismatch (infarct core volume <70 ml, ischemic penumbra-to-infarct core volume ratio ≥1.8, and mismatch volume ≥15 ml), as indicated by imaging, and 3) without endovascular treatment planned. The incidence of sICH within 36 h of treatment, the incidence of death from any cause, and the incidence of moderate-to-severe systemic hemorrhage were the safety endpoint events. The 516 included patients were randomly assigned in a 1:1 ratio to the TNK group (at a dose of 0.25 mg/kg, a maximum of 25 mg) and the conventional antiplatelet aggregation drug group. The primary endpoint event was the proportion of mRS scores of 0 to 1 at 90 days of onset. The TNK group was more effective than the conventional antiplatelet aggregation drug group (33.0% vs. 24.2%, *P* = 0.030); the two groups did not differ statistically in terms of safety, with the rate of symptomatic intracranial hemorrhage within 36 h of treatment being 3.0% vs. 0.8% and the rate of mortality at 90 days being 13.3% vs. 13.1%. Therefore, it can be said that this study discovered that if an ischemic penumbra is discovered following thorough imaging screening, treating TNK at this time can lower the rate of disability and greatly improve the functional outcome of patients with AIS who have onset between 4.5 and 24 h and combined bilateral internal carotid artery or middle cerebral artery M1 and M2 segment occlusion. The TRACE-3 study excluded patients with intended endovascular therapy, which may have decreased confounding factors for the effectiveness of intravenous thrombolysis and produced good results, in contrast to the TIMELESS study. In order to investigate the effectiveness of ultra-time window use of TNK in patients with basilar artery occlusion, the team is also presently working on a number of randomized controlled trials in the field of ultra-time window intravenous thrombolysis therapy. One such study is the ongoing TRACE-5 study (NCT06196320).

Patients with AIS within 24 h of the LKW time, intracranial anterior circulation large artery blockage, and perfusion CT showing a perfusion-infarct core mismatch were recruited for the Australian ETERNAL-LVO trial (44). Patients were randomized to either the rt-PA (0.90 mg/kg) or the usual medication therapy group, as well as the TNK (0.25 mg/kg) group. Prof. Vignan Yogendrakumar, the study's principal sponsor, presented the findings at this year's International Stroke Congress 2025. Both groups' primary functional outcome (the percentage of mRS scores of 0–1 at 90 days or return to baseline mRS levels) and the percentage of all-cause deaths at 90 days, the incidence of sICH, and other serious adverse events did not differ statistically significantly. The COVID-19 outbreak at the start of the trial caused it to end early, and due to the small sample size, it was not possible to show that TNK proved more advantageous than the current standard of care for patients with large vessel occlusion in the 0–24 h period (45).

The Chinese Stroke Association Guidelines for Reperfusion Therapy in Acute Ischemic Stroke 2024 (46) recommended intravenous thrombolysis with tenecteplase 0.25 mg/kg for AIS patients with the following conditions (Class I recommendation, Level A evidence), based on the positive outcomes of TRACE-3 and other studies:

① Internal carotid artery or middle cerebral artery M1, M2 segment occlusion and infarct core/hypoperfusion mismatch were confirmed by imaging examination.

② Endovascular thrombectomy was not possible.

③ Time of onset within 4.5–24 h and NIHSS score ≥6 after evaluation. The development of ultra-time window thrombolysis signifies the shift in AIS treatment from “time window” to “tissue window,” and more patients will have hope in the future thanks to the accurate use of imaging technology, the accumulation of TNK evidence-based medicine, and the enhancement of personalized treatment plans.

### 4.3 Ongoing AIS ultra-time window intravenous thrombolysis

Ultra-time window IVT is a forefront global research area. Numerous well-designed RCTs are underway internationally (Table 3), whose results are anticipated to refine clinical guidelines further and open new avenues for AIS treatment.

**Table 3:** Ongoing ultra-time-window intravenous thrombolysis studies.

**Research name (registration number)**	**Country**	**Time window (*h*)**	**Patient type**	**Intervention/ control**
POST-ETERNAL (NCT05105633)	Australia	0–24	BAO	TNK vs. SOC
OPTION (NCT05752916)	China	4.5–24	NON-LVO	TNK vs. SOC
RESILIENT EXTEND-IV (NCT05199662)	Brazil	4.5–12	NON-LVO	TNK vs. placebo
TNK-MeVO (NCT06559436)	China	4.5–24	MVO	TNK vs. SOC
TRACE-5 (NCT06196320)	China	4.5–24	BAO	TNK vs. SOC

POST-ETERNAL, Post-Eternal Study of Extended-Time-Window TNK IV Thrombolysis for Posterior Circulation Basilar Artery Occlusion Stroke; OPTION, OPTION Study of Extended-Time-Window TNK IV Thrombolysis for Acute Non-Large Vessel Occlusion Stroke; RESILIENT EXTEND-IV, Randomized Study of Extended Intravenous TNK Thrombolysis for Non-Large Vessel Occlusion Stroke; TNK-MeVO, TNK Intravenous Thrombolysis for Middle Vessel Occlusion Stroke Beyond the Time Window; TRACE-5, TNK Reperfusion Therapy for Acute Ischemic Cerebrovascular Events 5: Neurological Outcome Improvement with TNK Beyond the Time Window in Basilar Artery Occlusion; BAO, Basilar Artery Occlusion; NON-LVO, Non-Large Vessel Occlusion; MVO, Medium Vessel Occlusion; SOC, Standard of Care.

## 5 Summary and prospects

According to previous studies, individuals with AIS who receive IVT must complete the therapy within 4.5 h of onset. In addition to 14%−27% of patients with AIS whose time of onset is uncertain (e.g., waking strokes or unwitnessed strokes), the great majority of patients do not obtain timely intervention within the effective time window for thrombolysis (37, 47). Ultra-time window intravenous thrombolysis has become feasible in recent years due to the effective implementation of the multimodal imaging assessment techniques described in the aforementioned RCT studies. Further efforts are needed to address the dissociation between reperfusion and functional outcomes (e.g., the CHABLIS-T II study), the frequency of imaging screening in places with limited resources, and the delay in thrombolytic decision-making. Future research could examine more streamlined and precise dynamic imaging techniques for accurate patient stratification, the use of neuroprotective medications in conjunction with anti-inflammatory drugs to increase effectiveness, and international multicenter studies to confirm that TRACE-3 results are generalizable across ethnic and healthcare resource contexts.

## References

[1] GBD 2021 Diseases and Injuries Collaborators. Global incidence, prevalence, years lived with disability (YLDs), disability-adjusted life-years (DALYs), and healthy life expectancy (HALE) for 371 diseases and injuries in 204 countries and territories and 811 subnational locations, 1990–2021: a systematic analysis for the Global Burden of Disease Study 2021. Lancet. (2024) 403:2133–61. 38642570 10.1016/S0140-6736(24)00757-8 PMC11122111

[2] 《中国卒中中心报告2022》编写组 王陇德.《中国卒中中心报告2022》概要. 中国脑血管病杂志. (2024) 21:565–76. doi: 10.3969/j.issn.1672-5921.2024.08.009

[3] Tsao CW Aday AW Almarzooq ZI Anderson CAM Arora P Avery CL . Heart disease and stroke statistics-2023 update: a report from the American heart association. Circulation. (2023) 147:e93–621. doi: 10.1161/CIR.0000000000001137 36695182 PMC12135016

[4] Tu WJ Wang LD. Special writing group of China stroke surveillance report. China stroke surveillance report 2021. Mil Med Res. (2023) 10:33. doi: 10.1186/s40779-023-00463-x 37468952 PMC10355019

[5] 中华人民共和国国家卫生健康委员会. 脑血管病防治指南(2024年版). 磁共振成像. (2025) 16:1–8. doi: 10.5089/9798400267468.002

[6] Astrup J Siesjö BK Symon L. Thresholds in cerebral ischemia - the ischemic penumbra. Stroke. (1981) 12:723–25. doi: 10.1161/01.STR.12.6.723 6272455

[7] Ermine CM Bivard A Parsons MW Baron JC. The ischemic penumbra: from concept to reality. Int J Stroke. (2021) 16:497–509. doi: 10.1177/1747493020975229 33818215

[8] Yang SH Liu R. Four Decades of Ischemic Penumbra and Its Implication for ischemic stroke. Transl Stroke Res. (2021) 12:937–45. doi: 10.1007/s12975-021-00916-2 34224106 PMC8557134

[9] 方琪. 从时间窗到组织窗—-缺血性卒中治疗的机遇和挑战. 中国卒中杂志. (2020) 15:1025–27. doi: 10.3969/j.issn.1673-5765.2020.09.020

[10] Pan Y Shi G. Silver jubilee of stroke thrombolysis with alteplase: evolution of the therapeutic window. Front Neurol. (2021) 12:593887. doi: 10.3389/fneur.2021.593887 33732203 PMC7956989

[11] Vagal A Aviv R Sucharew H Reddy M Hou Q Michel P . Collateral clock is more important than time clock for tissue fate. Stroke. (2018) 49:2102–07. doi: 10.1161/STROKEAHA.118.021484 30354992 PMC6206882

[12] 中国卒中学会脑血流与代谢分会. 缺血性卒中脑侧支循环评估与干预中国指南 (2017). 中华内科杂志. (2017) 56:460–71. doi: 10.3760/cma.j.issn.0578-1426.2017.06.016

[13] Uniken Venema SM Dankbaar JW van der Lugt A Dippel DWJ van der Worp HB. Cerebral collateral circulation in the era of reperfusion therapies for acute ischemic stroke. Stroke. (2022) 53:3222–34. doi: 10.1161/STROKEAHA.121.037869 35938420

[14] Albers GW Marks MP Kemp S Christensen S Tsai JP Ortega-Gutierrez S . Thrombectomy for stroke at 6 to 16 h with selection by perfusion imaging. N Engl J Med. (2018) 378:708–18. doi: 10.1056/NEJMoa1713973 29364767 PMC6590673

[15] Nogueira RG Jadhav AP Haussen DC Bonafe A Budzik RF Bhuva P . Thrombectomy 6 to 24 h after stroke with a mismatch between deficit and infarct. N Engl J Med. (2018) 378:11–21. doi: 10.1056/NEJMoa1706442 29129157

[16] Ma H Campbell BCV Parsons MW Churilov L Levi CR Hsu C . Thrombolysis guided by perfusion imaging up to 9 h after onset of stroke. N Engl J Med. (2021) 384:1278. doi: 10.1056/NEJMx200014 33789024

[17] Xiong Y Campbell BCV Schwamm LH Meng X Jin A Parsons MW . Tenecteplase for ischemic stroke at 45 to 24 h without thrombectomy. N Engl J Med. (2024) 391:203–12. doi: 10.1056/NEJMoa2402980 38884324

[18] Nagai K Aoki J Sakamoto Y Kimura K. About 30% of wake-up stroke patients may be candidate for the tPA therapy using negative-FLAIR as a “tissue clock”. J Neurol Sci. (2017) 382:101–4. doi: 10.1016/j.jns.2017.09.042 29110999

[19] Nelson A Kelly G Byyny R Dionne C Preslaski C Kaucher K. Tenecteplase utility in acute ischemic stroke patients: a clinical review of current evidence. Am J Emerg Med. (2019) 37:344–48. doi: 10.1016/j.ajem.2018.11.018 30471930

[20] Gong L Liu M Zeng T Shi X Yuan C Andreasen PA . Crystal structure of the michaelis complex between tissue-type plasminogen activator and plasminogen activators inhibitor-1. J Biol Chem. (2015) 290:25795–804. doi: 10.1074/jbc.M115.677567 26324706 PMC4646234

[21] Logallo N Novotny V Assmus J Kvistad CE Alteheld L Rønning OM . Tenecteplase vs. alteplase for management of acute ischaemic stroke (NOR-TEST): a phase 3, randomised, open-label, blinded endpoint trial. Lancet Neurol. (2017) 16:781–88. doi: 10.1016/S1474-4422(17)30253-3 28780236

[22] Kvistad CE Næss H Helleberg BH Idicula T Hagberg G Nordby LM . Tenecteplase vs. alteplase for the management of acute ischaemic stroke in Norway (NOR-TEST 2, part A): a phase 3, randomised, open-label, blinded endpoint, non-inferiority trial. Lancet Neurol. (2022) 21:511–19. doi: 10.1016/S1474-4422(22)00124-7 35525250

[23] Katsanos AH Safouris A Sarraj A Magoufis G Leker RR Khatri P . Intravenous thrombolysis with tenecteplase in patients with large vessel occlusions: systematic review and meta-analysis. Stroke. (2021) 52:308–12. doi: 10.1161/STROKEAHA.120.030220 33272127

[24] Menon BK Buck BH Singh N Deschaintre Y Almekhlafi MA Coutts SB . Intravenous tenecteplase compared with alteplase for acute ischaemic stroke in Canada (AcT): a pragmatic, multicentre, open-label, registry-linked, randomised, controlled, non-inferiority trial. Lancet. (2022) 400:161–69. doi: 10.1016/S0140-6736(22)01054-6 35779553

[25] Wang Y Li S Pan Y Li H Parsons MW Campbell BCV . Tenecteplase vs. alteplase in acute ischaemic cerebrovascular events (TRACE-2): a phase 3, multicentre, open-label, randomised controlled, non-inferiority trial. Lancet. (2023) 401:1078. doi: 10.1016/S0140-6736(23)00627-X 36774935

[26] Meng X Li S Dai H Lu G Wang W Che F . Tenecteplase vs alteplase for patients with acute ischemic stroke: the ORIGINAL randomized clinical trial. JAMA. (2024) 332:1437–45. doi: 10.1001/jama.2024.14721 39264623 PMC11393753

[27] Muir KW Ford GA Ford I Wardlaw JM McConnachie A Greenlaw N . Tenecteplase vs. alteplase for acute stroke within 4·5 h of onset (ATTEST-2): a randomised, parallel group, open-label trial. Lancet Neurol. (2024) 23:1087–96. doi: 10.1016/S1474-4422(24)00377-6 39424558

[28] Parsons MW Yogendrakumar V Churilov L Garcia-Esperon C Campbell BCV Russell ML . Tenecteplase vs. alteplase for thrombolysis in patients selected by use of perfusion imaging within 4.5 h of onset of ischaemic stroke (TASTE): a multicentre, randomised, controlled, phase 3 non-inferiority trial. Lancet Neurol. (2024) 23:775–86. doi: 10.1016/S1474-4422(24)00206-0 38880118

[29] Xiong Y Wang L Li G Yang KX Hao M Li S . Tenecteplase vs. alteplase for acute ischaemic stroke: a meta-analysis of phase III randomised trials Stroke. Vasc Neurol. (2024) 9:360–66. doi: 10.1136/svn-2023-002396 PMC11420912 37640500

[30] Warach SJ Ranta A Kim J Song SS Wallace A Beharry J . Symptomatic intracranial hemorrhage with tenecteplase vs alteplase in patients with acute ischemic stroke: the comparative effectiveness of routine tenecteplase vs alteplase in acute ischemic stroke (CERTAIN) collaboration. JAMA Neurol. (2023) 80:732–38. doi: 10.1001/jamaneurol.2023.1449 37252708 PMC10230371

[31] Liu Y Lu G Li D Wu G Zhou X Qu R . Tenecteplase thrombolytic therapy for acute ischaemic stroke in China: a real-world, multicentre, retrospective, controlled study. Stroke Vasc Neurol. (2025) 10:452–61. doi: 10.1136/svn-2024-003381 39537238 PMC12415637

[32] Warner JJ Harrington RA Sacco RL Elkind MSV. Guidelines for the early management of patients with acute ischemic stroke: 2019 update to the 2018 guidelines for the early management of acute ischemic stroke. Stroke. (2019) 50:3331–2. doi: 10.1161/STROKEAHA.119.027708 31662117

[33] Berge E Whiteley W Audebert H De Marchis GM Fonseca AC Padiglioni C . European Stroke Organisation (ESO) guidelines on intravenous thrombolysis for acute ischaemic stroke. Eur Stroke J. (2021) 6:I-LXII. doi: 10.1177/2396987321989865 33817340 PMC7995316

[34] 中国医师协会神经内科医师分会脑血管病专家组 陈会生 杨清武 等.急性缺血性卒中替奈普酶静脉溶栓治疗中国专家共识. 中国神经精神疾病杂志. (2022) 48:641–51. doi: 10.3969/j.issn.1002-0152.2022.11.001

[35] Crow J Smith A. National clinical guideline for stroke for the United Kingdom and Ireland: Part I - an overview of the updated recommendations. Br J Occup Ther. (2023) 86:661–64. doi: 10.1177/03080226231188020 40337193 PMC12033436

[36] 中华医学会神经病学分会 中华医学会神经病学分会脑血管病学组.中国急性缺血性卒中诊治指南2023. 中华神经科杂志. (2024) 57:523–59. doi: 10.3760/cma.j.cn113694-20240410-00221

[37] Thomalla G Simonsen CZ Boutitie F Andersen G Berthezene Y Cheng B . MRI-guided thrombolysis for stroke with unknown time of onset. N Engl J Med. (2018) 379:611–22. doi: 10.1056/NEJMoa1804355 29766770

[38] Cheng B Boutitie F Nickel A Wouters A Cho TH Ebinger M . Quantitative signal intensity in fluid-attenuated inversion recovery and treatment effect in the WAKE-UP trial. Stroke. (2020) 51:209–15. doi: 10.1161/STROKEAHA.119.027390 31662118

[39] Thomalla G Boutitie F Ma H Koga M Ringleb P Schwamm LH . Intravenous alteplase for stroke with unknown time of onset guided by advanced imaging: systematic review and meta-analysis of individual patient data. Lancet. (2020) 396:1574–84. doi: 10.1016/S0140-6736(20)32163-2 33176180 PMC7734592

[40] Roaldsen MB Eltoft A Wilsgaard T Christensen H Engelter ST Indredavik B . Safety and efficacy of tenecteplase in patients with wake-up stroke assessed by non-contrast CT (TWIST): a multicentre, open-label, randomised controlled trial. Lancet Neurol. (2023) 22:117–26. doi: 10.1016/S1474-4422(22)00484-7 36549308

[41] Albers GW Jumaa M Purdon B Zaidi SF Streib C Shuaib A . Tenecteplase for stroke at 45 to 24 h with perfusion-imaging selection. N Engl J Med. (2024) 390:701–11. doi: 10.1056/NEJMoa2310392 38329148

[42] Leifer D. Tenecteplase for stroke - opening the window? N Engl J Med. (2024) 390:760–61. doi: 10.1056/NEJMe2314930 38329103

[43] Wang L Dai YJ Cui Y Zhang H Jiang CH Duan YJ . Intravenous tenecteplase for acute ischemic stroke within 45–24 h of onset (ROSE-TNK): a phase 2, randomized, multicenter study. J Stroke. (2023) 25:371–77. doi: 10.5853/jos.2023.00668 37608533 PMC10574303

[44] Yogendrakumar V Campbell BC Churilov L Garcia-Esperon C Choi PM Cordato DJ . Extending the time window for tenecteplase by effective reperfusion of penumbral tissue in patients with large vessel occlusion: rationale and design of a multicenter, prospective, randomized, open-label, blinded-endpoint, controlled phase 3 trial. Int J Stroke. (2025) 20:367–72. doi: 10.1177/17474930241308660 39654273 PMC11874470

[45] Yogendrakumar V Campbell BCV Churilov L Garcia-Esperon C Choi PMC Cordato DJ . Efficacy of tenecteplase in large vessel occlusion stroke within 24 hours of symptom onset: the ETERNAL-LVO randomized controlled trial. Stroke. (2025) 56:3332–41. doi: 10.1161/STROKEAHA.125.052511 40927857

[46] 中国卒中学会. 《中国卒中学会急性缺血性卒中再灌注治疗指南》编写组, 王拥军. 中国卒中学会急性缺血性卒中再灌注治疗指南2024. 中国卒中杂志. (2024) 19:1460–78. doi: 10.3969/j.issn.1673-5765.2024.12.014

[47] Kim YJ Kim BJ Kwon SU Kim JS Kang DW . Unclear-onset stroke: daytime-unwitnessed stroke vs. wake-up stroke. Int J Stroke. (2016) 11:212–20. doi: 10.1177/1747493015616513 26783313

[48] Cheng X Hong L Churilov L Lin L Ling Y Zhang J . Tenecteplase thrombolysis for stroke up to 24 h after onset with perfusion imaging selection: the umbrella phase IIa CHABLIS-T randomised clinical trial. Stroke Vasc Neurol. (2024) 9:551–59. doi: 10.1136/svn-2023-002820 38286484 PMC11732838

[49] Coutts SB Ankolekar S Appireddy R Arenillas JF Assis Z Bailey P . Tenecteplase vs. standard of care for minor ischaemic stroke with proven occlusion (TEMPO-2): a randomised, open label, phase 3 superiority trial. Lancet. (2024) 403:2596. doi: 10.1016/S0140-6736(24)00921-8 38768626

[50] Cheng X Hong L Lin L Churilov L Ling Y Yang N . Tenecteplase thrombolysis for stroke up to 24 h after onset with perfusion imaging selection: the CHABLIS-T II randomized clinical trial. Stroke. (2025) 56:344–54. doi: 10.1161/STROKEAHA.124.048375 39744861

